# Management of a Complex Supracondylar Periprosthetic Femur Fracture with Intramedullary Strut Allograft and Bilateral Locking Plates

**DOI:** 10.1155/2021/4882382

**Published:** 2021-11-17

**Authors:** Rita Moukarzel, Dany Aouad, Mohammad Daher, Wendy Ghanem, Hady Ezzeddine, George El Rassi

**Affiliations:** ^1^Medical School, Lebanese American University Medical Center, Lebanese American University, P.O. Box 13-5053, Chouran Beirut: 1102 2801, Lebanon; ^2^Department of Orthopedic Surgery and Traumatology, Saint Georges University Medical Center, Balamand University, P.O.Box 166378, Achrafieh, Beirut 1100 2807, Lebanon; ^3^Faculty of Medicine, University Saint Joseph, P.O. Box 17-5208, Mar Mikhael, Beirut, Lebanon

## Abstract

Supracondylar periprosthetic femoral fractures occurring above total knee replacements have been considered a rare entity. However, they continue to increase in frequency with the increasing number of arthroplasties and the improvement in morbidity and mortality in the concerned patient population. The management of periprosthetic distal femoral fractures is a challenging orthopedic problem. In this brief communication, a case of 49-year-old woman with rheumatoid arthritis who sustained a low distal comminuted periprosthetic femoral fracture is presented. Her fracture was eventually managed with an intramedullary fibular strut allograft and bilateral locking plate placement reaching satisfactory healing and restoration of alignment. The primary aim of this report is to provide insight into this novel technique as a successful alternative to other standard surgical options.

## 1. Introduction

Supracondylar periprosthetic femoral fractures have been considered a rare complication of total knee replacements. However, it is no surprise that they continue to increase in frequency alongside the rising number of total knee arthroplasties performed yearly and the improved quality of life and life expectancy of the concerned patient population [1].

These fractures generally occur in the geriatric population aged above 60 years with associated osteopenic or osteoporotic bone quality [2]. Commonly, the fracture is secondary to minimal low-velocity trauma [3]. In the setting of total knee arthroplasties, the supracondylar distal femur is the most frequent location for periprosthetic fractures [4], which are specifically associated with comminution [5]. Soininvaara et al. reported up to 25.5% rapid bone loss in the distal femoral bone in the first 6-month period posttotal knee arthroplasty [6]. In fact, management with bisphosphonates after total knee arthroplasty helps decrease the periprosthetic osteopenia [7].

Distal periprosthetic femoral fractures can be managed either conservatively or surgically. Nevertheless, they are all managed surgically in patients tolerable of anesthesia who are otherwise ambulatory in order to prevent the complications of conservative management [8].

This review shares the management of a complicated periprosthetic distal femoral fracture with a novel combination surgical technique that uses an intramedullary fibular strut allograft supplemented with bilateral plate-and-screw placement. This method was used on a 49-year-old woman, with rheumatoid arthritis, who sustained a low distal comminuted periprosthetic femoral fracture about a total knee arthroplasty (TKA). The fracture was initially managed with surgical open reduction and internal fixation (ORIF) with lateral plate placement but failed to achieve appropriate union and alignment. Ultimately, the fracture was successfully managed with an intramedullary fibular strut allograft with bilateral locking plate placement.

The effective use of retrograde intramedullary fibular strut allograft is proposed to augment plate fixation especially when the distal portion is small for instrument insertion or when the bone stock is low with increased comminution. The primary aim of this article is to provide insight into this novel surgical technique as a satisfactory alternative to traditional treatment options for managing periprosthetic distal femoral fractures, particularly in osteopenic patients.

## 2. Case Report

This is a case of a 49-year-old female patient, known to have juvenile rheumatoid arthritis currently managed with methotrexate and adalimumab. The patient had undergone a bilateral total knee replacement ten years prior to presentation.

**Figure 1:**
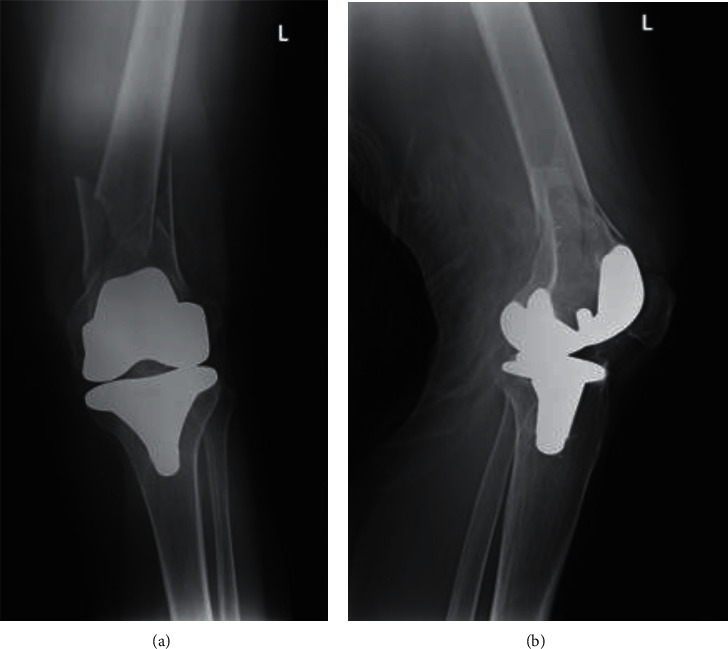
Preoperative A-P (a) and lateral (b) radiographs showing a complex left supracondylar distal femoral fracture adjacent to a total knee arthroplasty.

She initially presented after sustaining a low-energy fall from standing height. Plain radiographs were done and showed a supracondylar displaced periprosthetic fracture of the left distal femur (Figure 1).

**Figure 2:**
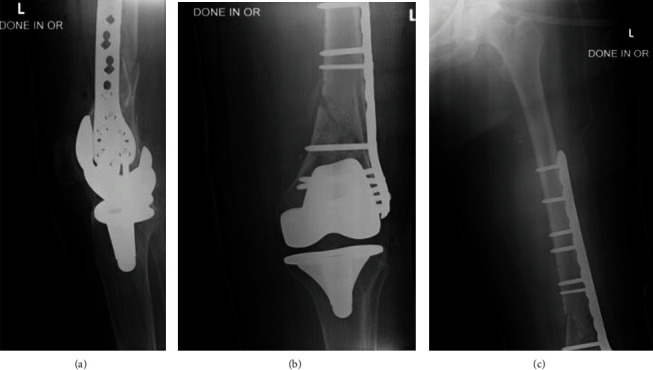
Postoperative lateral (a) and A-P (b, c) radiographs done in the OR, showing reduction of fracture status post management with ORIF and lateral plate-and-screw fixation.

The fracture was comminuted with segmental bone defect in the distal femoral region. The patient underwent surgical open reduction and internal fixation (ORIF) with bone graft and lateral plate-and-screw (Figure 2).

**Figure 3:**
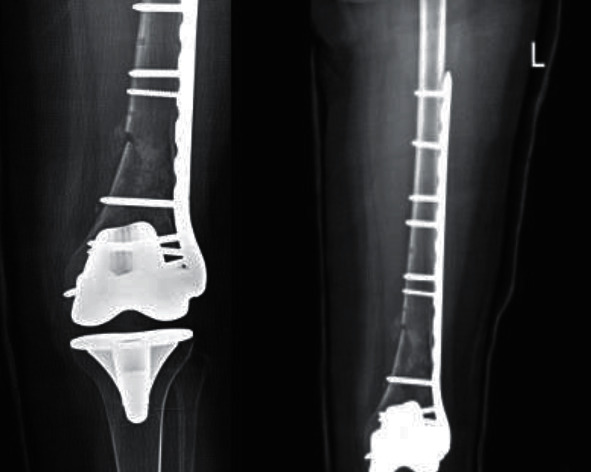
Postoperative follow-up A-P radiographs done one month postoperatively showing reduced fracture and beginning of callus formation in the distal femoral region.

The surgery was performed with neither intraoperative nor direct postoperative complications, and the patient was discharged one week postoperatively. The patient was followed with a series of radiographs to assess for satisfactory healing, alignment, and stable construct fixation. One month postoperatively, a follow-up radiograph showed fracture reduction with start of callus formation (Figure 3).

**Figure 4:**
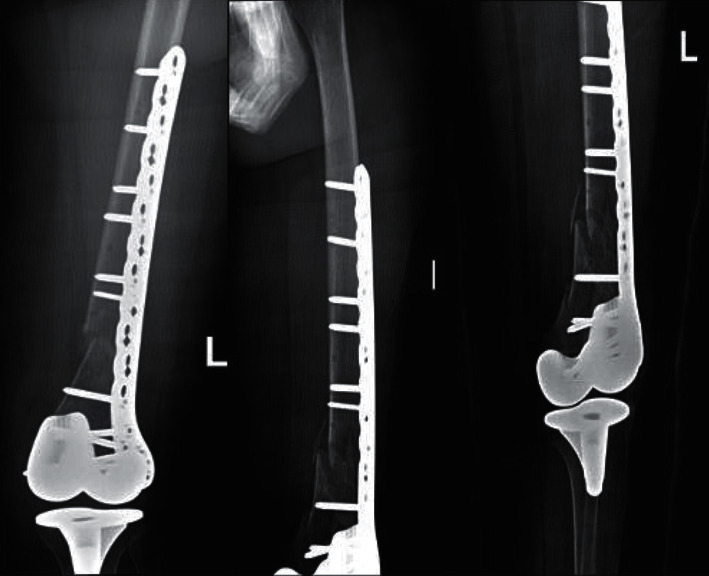
Postoperative follow-up A-P and lateral radiographs done two months postoperatively showing nonunion with severe comminution in the distal femoral region which is also associated with low bone stock.

**Figure 5:**
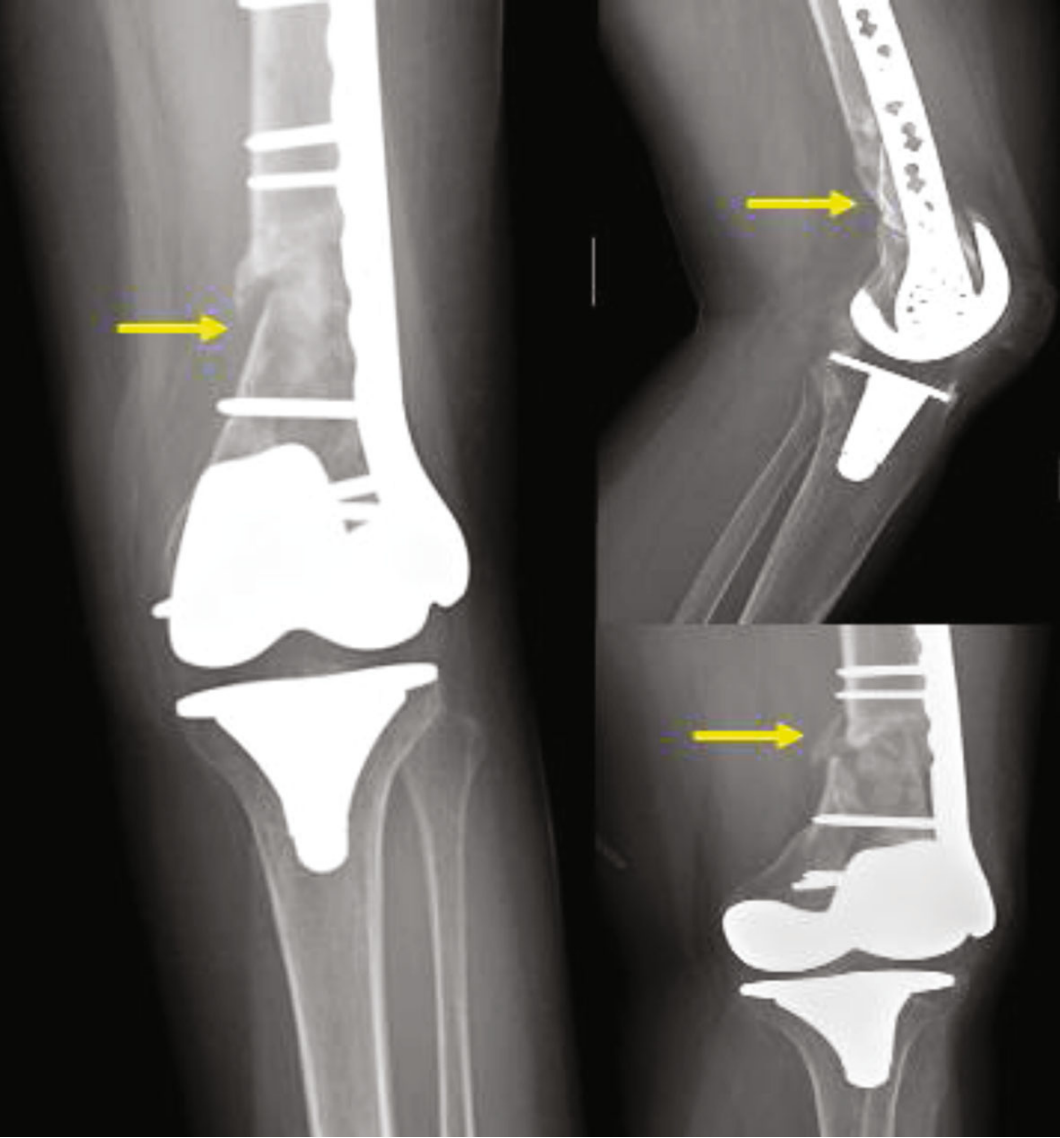
Postoperative follow-up AP and lateral radiographs done nine months after the surgery still showing nonunion, comminution, and low bone stock (yellow arrows).

Two months postoperatively, follow-up radiographs (Figure 4) showed nonunion with severe comminution in the distal femoral region characterized by significantly low bone stock, which was also seen in the radiographs that followed in seven and nine months postoperatively (Figure 5).

**Figure 6:**
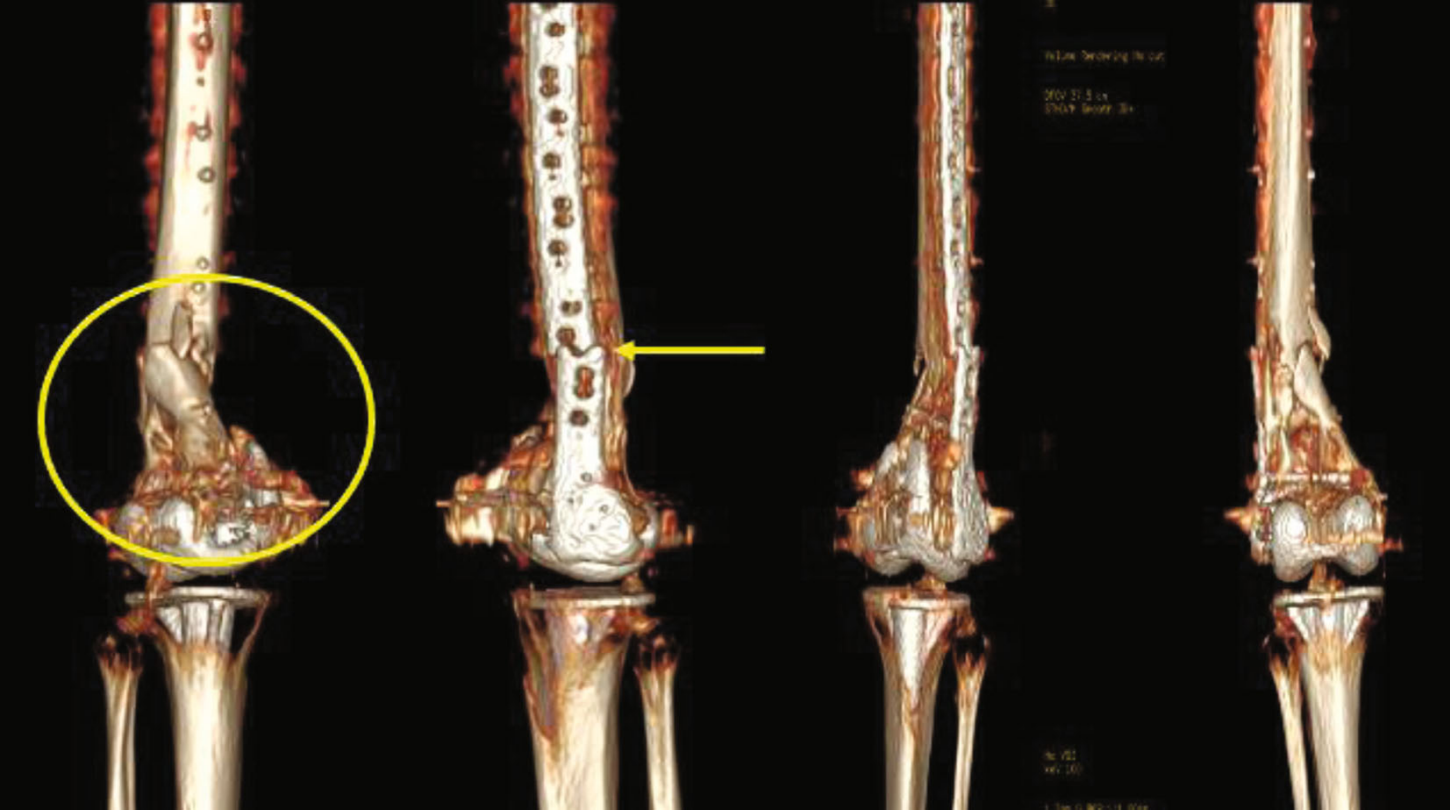
Postoperative follow-up with 3D reconstruction done one year postoperatively showing fracture nonunion (yellow circle), malalignment, and plate breakage (yellow arrow).

A computed tomography scan with 3D reconstruction was done one year after the surgery showing failure of hardware, nonunion, and malalignment (Figure 6).

Consequently, an elective surgical repair was scheduled. The primary lateral plate was removed, and an intramedullary fibular allograft was introduced through the intercondylar region into the medullary cavity supplemented with lateral plate fixation (lateral curved LCP 8 hole condylar plate (Synthes, Beirut, Lebanon)) and medial minimally invasive (MIS) plate (medial curved LCP 18 hole condylar plate (Synthes, Beirut Lebanon)) fixation. Demineralized bone matrix (DBM) and bone morphogenic protein-2 (BMP-2) were also inserted.

**Figure 7:**
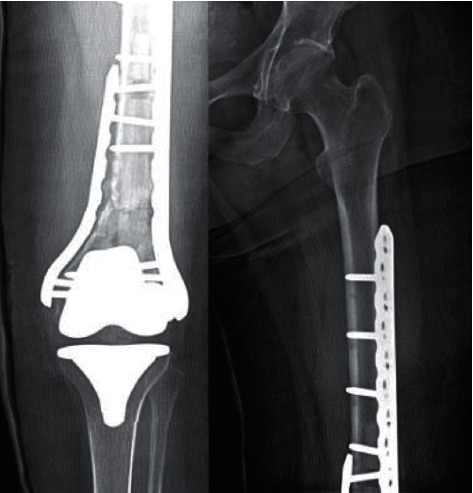
Postoperative follow-up radiographs done one month after revision surgery showing satisfactory alignment.

Follow-up radiographs (Figure 7) done one month postoperatively showed stable fixation and alignment with beginning of healing and callus formation.

**Figure 8:**
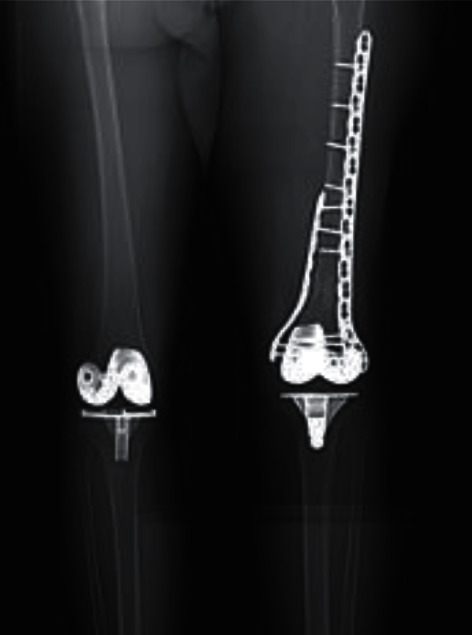
Postoperative follow-up AP radiograph of both knees nine months after revision surgery showing improvement in healing and satisfactory alignment.

In addition, further follow-up imaging done at three, five, and seven months postoperatively (Figure 8) showed improvement in healing.

The patient progressively improved to a painless, full range of motion mobilization of the knee. She suffered no postoperative complications of graft rejection, infection, or mechanical instability on weightbearing.

**Figure 9:**
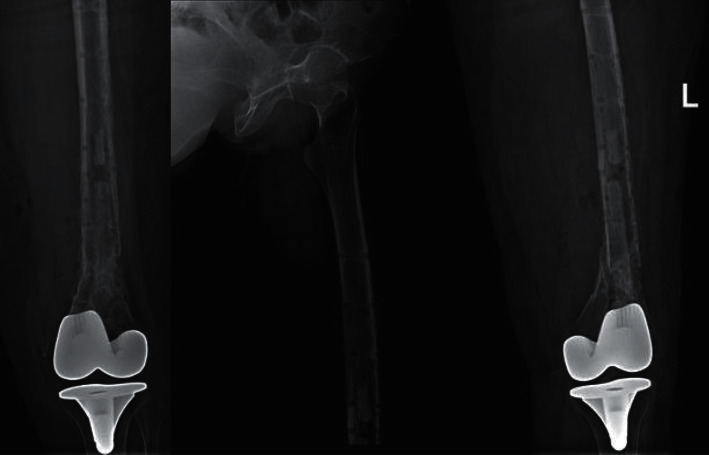
Postoperative radiographs two months after hardware removal showing complete healing of the fracture with satisfactory alignment.

Ten months after the surgery, the patient was admitted for bilateral plate removal with bone graft placed in screw holes. Cultures obtained two months later revealed negative tissue and serum results, and plain radiographs (Figure 9) showed complete healing of the fracture with a satisfactory alignment.

## 3. Discussion

Supracondylar periprosthetic femoral fractures occur usually in geriatric populations [2] due to low velocity traumas such as a fall from standing height [3]. Osteoporotic and osteopenic bone is the major risk factors for such injuries [2]. Other predisposing factors include female gender, rheumatoid arthritis, chronic steroid use, neurological diseases and recurrent falls, intraoperative anterior femoral notching, and revision arthroplasty [1, 3], among which risk factors leading to low bone density considered more important [4].

Prognostically, periprosthetic femoral shaft fractures are associated with better postoperative outcomes compared to periprosthetic distal femoral fractures which carry high postoperative morbidity and mortality [1]. Hence, periprosthetic distal femoral fractures are technically challenging to manage, particularly in elderly osteopenic patients with associated thin cortices and loss of bone stock [9]. The deficient bone density in the distal femoral region as well as the low fracture localization adjacent to an arthroplasty component [2] obligates the modification of traditional fixation techniques. Specifically, low fractures with a little distal osteopenic fragment would impede strong fixation [10].

Although the management of these fractures has been increasingly widely practiced, postoperative complications continue to be high in these patients regardless of the technique used. These include reduced knee range of motion, residual fracture malunion, nonunion, malalignment, infection, and perioperative death [3]. Therefore, the treatment goals must comprise painless healing, early restoration of range of motion and weight-bearing, uncomplicated fracture union, radiographic alignment restoration, and return to preinjury function [1]. Reestablishing appropriate bone stock and ensuring prosthesis mechanical stability are also essential considerations to safeguard treatment success [1].

Preoperative evaluation must take into account the anatomic fracture site with respect to the arthroplasty component, prosthesis stability and type, local bone stock, bone displacement, and patient's preinjury ambulatory status and past medical history [1, 3] in order to decide on the most suitable management technique.

This injury can both be managed conservatively and surgically. Conservative treatment primarily entails cast immobilization and is usually reserved for undisplaced fracture types [2]. Nonconservative surgical treatment options include open reduction and internal fixation (ORIF), external fixation, anterograde or retrograde intramedullary nailing, internal fixation with locking plates, and distal femoral replacement, among others [5]. The latter must be considered after considering all treatment options due to its disastrous complications [11].

Surgical management is considered superior to conservative treatment because the latter is associated with prolonged immobilization and carries increased risks of nonunion and reoperation [2]. Although nonsurgical management avoids perioperative complications, Moran et al. mentions that 12-40% of the cases managed nonoperatively resulted in nonunion, and 15-30% of cases required reoperation [12]. Also, progression from nondisplaced to displaced fractures ensued in many cases, which required close radiologic follow-up [2]. Therefore, in most cases, orthopedists opt for surgical fixation as the primary option for managing these fractures. In most cases, ORIF with conventional plate fixation is performed as it presents a safe and minimally invasive surgical technique. In cases where the fracture is close to the arthroplasty component anteriorly, retrograde intramedullary nail combined with plate-and-screw fixation is used [8]. However, in cases like the one presented above, where the fracture is too distal and the metaphyseal region is severely comminuted and osteopenic for plate placement or nail insertion, using an intramedullary bulk allograft can be of aid. The intramedullary graft provides stable reduction and fixation for subsequent plate insertion. A multicenter experience by Rollo et al. showed that a combination of both strut allografts and plating may be the most efficient method of treatment of femoral periprosthetic fractures [13]. It is even more efficient than the treatment with minimally invasive plate osteosynthesis [14].

The aim of treating a periprosthetic fracture above TKA and severe osteopenia in a weightbearing bone must primarily target mechanical stability of the fixation construct. Intraoperatively, the fibular allograft was initially introduced into the distal femoral medullary cavity through the intercondylar notch at the arthroplasty construct [5]. This ensured adequate fixation of the fracture for placement of the lateral locking plate and the minimally invasive medial MIS plate. This technique, however, is only performed by highly skilled orthopedic surgeons able to manipulate allografts for major reconstruction [5].

Ultimately, although this surgical technique presents prognostic and mechanical advantages to overcome these fractures, it certainly is not free of limitations. Just like any other operation, fracture reduction does carry with it a risk of nonunion, malalignment, and infection. Moreover, the use of an allograft might present with graft complications, like host-graft rejection and disease transmission, for which one must safeguard appropriate consideration [15].

## 4. Conclusion

In conclusion, patients presenting with a low periprosthetic distal femoral fracture in the setting of severe osteopenia and comminution should be assessed promptly for displacement, anatomic site of fracture, and arthroplasty component in order to ensure the management technique with the best outcome. Using an intramedullary fibular strut allograft is believed to augment the stability supplemented by bilateral plate insertion, and that it is a feasible alternative in managing these complicated fractures. The proposed technique is worth putting into practice as it provides good postoperative outcomes and improved quality of life in the concerned patient population.

## Data Availability

The data used to support the findings of this study are available from the corresponding author upon request.
